# Onchocerca parasites and *Wolbachia *endosymbionts: evaluation of a spectrum of antibiotic types for activity against *Onchocerca gutturosa in vitro*

**DOI:** 10.1186/1475-2883-5-4

**Published:** 2006-03-24

**Authors:** Simon Townson, Senyo Tagboto, Helen F McGarry, Gillian L Egerton, Mark J Taylor

**Affiliations:** 1 Tropical Parasitic Diseases Unit, Northwick Park Institute for Medical Research, Watford Road, Harrow, Middlesex HA1 3UJ, UK; 2 Filariasis Research Laboratory, Molecular and Biochemical Parasitology, Liverpool School of Tropical Medicine, Pembroke Place, Liverpool L3 5QA, UK

## Abstract

**Background:**

The filarial parasites of major importance in humans contain the symbiotic bacterium *Wolbachia *and recent studies have shown that targeting of these bacteria with antibiotics results in a reduction in worm viability, development, embryogenesis, and survival. Doxycycline has been effective in human trials, but there is a need to develop drugs that can be given for shorter periods and to pregnant women and children. The World Health Organisation-approved assay to screen for anti-filarial activity *in vitro *uses male *Onchocerca gutturosa*, with effects being determined by worm motility and viability as measured by reduction of MTT to MTT formazan. Here we have used this system to screen antibiotics for anti-filarial activity. In addition we have determined the contribution of *Wolbachia *depletion to the MTT reduction assay.

**Methods:**

Adult male *O. gutturosa *were cultured on a monkey kidney cell (LLCMK 2) feeder layer in 24-well plates with antibiotics and antibiotic combinations (6 to 10 worms per group). The macrofilaricide CGP 6140 (Amocarzine) was used as a positive control. Worm viability was assessed by two methods, (i) motility levels and (ii) MTT/formazan colorimetry. Worm motility was scored on a scale of 0 (immotile) to 10 (maximum) every 5 days up to 40 days. On day 40 worm viability was evaluated by MTT/formazan colorimetry, and results were expressed as a mean percentage reduction compared with untreated control values at day 40. To determine the contribution of *Wolbachia *to the MTT assay, the MTT formazan formation of an insect cell-line (C6/36) with or without insect *Wolbachia *infection and treated or untreated with tetracycline was compared.

**Results:**

Antibiotics with known anti-*Wolbachia *activity were efficacious in this system. Rifampicin (5 × 10^-5^M) was the most effective anti-mycobacterial agent; clofazimine (1.25 × 10^-5^M and 3.13 × 10^-6^M) produced a gradual reduction in motility and by 40 days had reduced worm viability. The other anti-mycobacterial drugs tested had limited or no activity. Doxycycline (5 × 10^-5^M) was filaricidal, but minocycline was more effective and at a lower concentration (5 × 10^-5^M and 1.25 × 10^-5^M). Inactive compounds included erythromycin, oxytetracycline, trimethoprim and sulphamethoxazole. The MTT assay on the insect cell-line showed that *Wolbachia *made a significant contribution to the metabolic activity within the cells, which could be reduced when they were exposed to tetracycline.

**Conclusion:**

The *O. gutturosa *adult male screen for anti-filarial drug activity is also valid for the screening of antibiotics for anti-*Wolbachia *activity. In agreement with previous findings, rifampicin and doxycycline were effective; however, the most active antibiotic was minocycline. *Wolbachia *contributed to the formation of MTT formazan in the MTT assay of viability and is therefore not exclusively a measure of worm viability and indicates that *Wolbachia *contributes directly to the metabolic activity of the nematode.

## Background

The control of human filariasis caused by *Brugia malayi*, *Wuchereria bancrofti *and *Onchocerca volvulus*, currently relies on community-wide mass distribution of ivermectin and albendazole, either individually or in combination with diethylcarbamazine. Unfortunately, these drugs are mainly microfilaricidal rather than macrofilaricidal, which means that repeated treatment is required over many years, and the possibility that resistance to them may develop is a cause for concern [1-3]. However, with the realisation that many filarial nematodes, including those responsible for most of the global burden of human filarial disease, are infected with *Wolbachia *[4], has provided an alternative approach to the treatment and control of these parasites with antibiotics.

*Wolbachia *are intracellular bacteria that have a mutualistic relationship with their nematode hosts [4] and studies have shown that antibiotics can eliminate the bacteria and result in worm growth retardation, infertility and reduced viability [5-8]; conversely, nematodes not infected with *Wolbachia *were unaffected by antibiotic exposure [5]. Recent field trials of the antibiotics oxytetracycline against onchocerciasis in cattle [9] and of doxycycline in human onchocerciasis [10-12] and *W. bancrofti *infection [13,14] have demonstrated the validity of this approach. In all cases, treatment resulted in a pronounced reduction in microfilaraemia/microfilaridermia and a prolonged inhibition of embryogenesis, but, significantly, an eight week course of doxycycline 200 mg daily also proved to be macrofilaricidal against *W. bancrofti *[13]. The administration of antibiotics may also have benefits in addition to their sterilising effects and killing of worms; *Wolbachia *are associated with the pathology of filarial infections [15-17] and with adverse reactions to diethylcarbamazine and ivermectin [18,19]. A recent study has shown that a three week course of doxycycline can lead to a sustained amicrofilaraemia but does not result in macrofilaricidal activity [20].

Although antibiotics have a valuable activity against filarial nematodes, the long treatment regimens that are required present logistical problems for mass drug administration (MDA). Another obstacle to MDA is the contra-indication of tetracyclines in pregnant women and children under the age of eight years. Therefore, alternative treatment options that target *Wolbachia *but circumvent these problems would be advantageous. For more than 18 years, an *in vitro *drug screen for identifying potential macrofilaricidal activity has used adult male *O. gutturosa *[21-23], with assessment of efficacy being made by observing worm motility and inhibition of MTT formazan formation [24,25]. In the present work we have extended this system and developed a long-term assay, which has enabled us to screen many of the commonly used antibiotics for macrofilaricidal activity, both individually and in combinations. We have also determined the contribution of *Wolbachia *to the MTT assay.

## Methods

### *In vitro *drug screen

Adult male *O. gutturosa *were dissected from the nuchal ligament connective tissues obtained from naturally infected cattle in Kumasi, Ghana, as previously described [6]. They were maintained individually in the wells of a 24-well plate containing 1.8 ml of Minimum Essential Medium with 10% heat inactivated calf serum and a monkey kidney cell (LLCMK2) feeder layer [26] at 36.5°C with 5% CO_2 _for 24 to 48 hours until the addition of drugs. The medium of all wells included the antibiotics penicillin (200 U/ml) and streptomycin (200 μg/ml), which have no anti-*Wolbachia *activity [27], and the anti-mycotic agent amphotericin B (50 μg/ml). Drugs were prepared as previously described [6] in medium and each drug concentration was tested against six to ten worms, maintained as above. Medium (with or without drug) was replaced every 5 days. The compounds tested included a range of test antibiotics (Tables 1, 2 and 3), both individually and in combination. The amoscanate derivative CGP 6140 (Amocarzine) was used as a positive control since it is macrofilaricidal against *Onchocerca *parasites [28], reviewed by [29].

**Table 1:** Summary of long-term trial 1: parasite mean motility scores and viability (MTT colorimetry)

**Compound/drug and conc.**	**Mean motility scores over a period of 40 days**									**Motility**	**MTT**
DAY	1	5	10	15	20	25	30	35	40	% reduction on day 40 (*P*)	% inhibition on day 40 (*P*)
Control	8.0	7.4	7.1	7.0	5.3	5.5	6.3	6.4	4.9		
CGP 6140 1.25 × 10^-5^M (positive control)	0.5	0.8	0.0	0.5	0.0	0.0	0.0	0.0	0.0	100.0 (0.005)	83.3 (0.098)
Rifampicin 5 × 10^-5^M	7.7	6.8	6.7	6.8	3.2	1.7	0.2	0.2	0.0	100.0 (0.002)	89.6 (0.033)
Rifampicin 1.25 × 10^-5^M	7.0	7.0	7.3	5.7	6.5	5.2	5.7	5.0	5.6	0.0 (0.647)	0.0 (0.798)
Minocycline 5 × 10^-5^M	8.0	6.7	7.3	5.7	0.0	0.0	0.0	0.0	0.0	100.0 (0.002)	84.1 (0.042)
Minocycline 1.25 × 10^-5^M	7.8	7.5	6.7	5.7	4.3	2.5	4.8	1.7	0.0	100.0 (0.002)	93.7 (0.026)
Doxycycline 5 × 10^-5^M	8.3	8.0	8.0	5.7	0.3	0.0	0.0	0.0	0.0	100.0 (0.002)	93.0 (0.027)
Doxycycline 1.25 × 10^-5^M	7.3	6.5	6.3	4.8	6.3	6.3	6.2	6.2	5.5	0.0 (0.699)	0.0 (0.801)
Ethambutol 5 × 10^-5^M	6.8	6.8	6.5	6.7	4.0	4.8	4.3	5.0	2.7	44.9 (0.170)	73.0 (0.108)
Dapsone 5 × 10^-5^M	7.3	7.3	7.7	5.0	6.8	7.0	7.2	7.3	6.5	0.0 (0.250)	0.0 (0.798)
Pyrazinamide 5 × 10^-5^M	7.7	7.7	7.2	3.5	5.5	5.5	5.7	5.2	3.7	24.5 (0.503)	4.8 (0.949)

The effects of exposure to a range of antibiotics on the viability of *Onchocerca* gutturosa adult males in long-term (40 day) *in vitro* culture. Viability was assessed by measuring worm motility scores throughout the trial and by MTT colorimetry on day 40.

**Table 2:** Summary of long-term trial 2: parasite mean motility scores and viability (MTT colorimetry)

**Compound/drug and conc.**	**Mean motility scores over a period of 40 days**									**Motility**	**MTT**
DAY	1	5	10	15	20	25	30	35	40	% reduction on day 40 (*P*)	% inhibition on day 40 (*P*)

Control	7.6	7.2	7.1	6.8	5.9	4.9	5.8	6.1	6.3		
^a^CGP 6140 1.25 × 10^-5^M (positive control)	1.3	0.0	0.0	0.0	0.0	0.0	0.0	0.0	0.0	100.0 (ND)	87.2 (ND)
^b^Norfloxacin 5 × 10^-5^M	6.9	7.4	7.1	2.4	2.5	2.6	1.4	1.0	2.5	60.3 (ND)	90.7 (ND)
Ciprofloxacin 5 × 10^-5^M	6.9	7.9	7.1	3.6	4.1	3.0	2.3	2.5	3.0	52.4 (0.080)	73.3 (0.040)
Vancomycin 5 × 10^-5^M	6.4	7.4	6.5	3.6	3.6	2.8	2.4	1.6	2.1	66.7 (0.009)	83.7 (0.022)
Gentamycin 5 × 10^-5^M	7.6	5.5	6.3	2.8	2.6	0.8	1.5	1.0	1.6	74.6 (0.005)	83.7 (0.017)
Ceftriaxone 5 × 10^-5^M	7.1	6.8	7.0	2.4	2.0	0.9	0.6	0.6	0.7	88.9 (<0.001)	90.7 (0.011)
^c^Triclosan 5 × 10^-5^M	6.9	4.9	0.4	0.1	0.0	0.0	0.0	0.0	0.0	100.0 (ND)	98.8 (ND)
^c^Cerulenin 5 × 10^-5^M	0.0	0.0	0.0	0.0	0.0	0.0	0.0	0.0	0.0	100.0 (ND)	100.0 (ND)

a. Day 40 results based on 1 worm; 5 worms lost to microbial contamination

b. Day 40 results based on 2 worms; 4 worms lost to microbial contamination

c. Denotes compounds that were toxic to the monkey kidney cell feeder layer

The effects of exposure to a range of antibiotics on the viability of *Onchocerca gutturosa *adult males in long-term (40 day) *in vitro *culture. Viability was assessed by measuring worm motility scores throughout the trial and by MTT colorimetry on day 40.

**Table 3:** Summary of long-term trial 3: parasite mean motility scores and viability (MTT colorimetry)

**Compound/drug and conc.**	**Mean motility scores over a period of 40 days**									**Motility**	**MTT**
DAY	1	5	10	15	20	25	30	35	40	% reduction on day 40 (*P*)	% inhibition on day 40 (*P*)
Control	8.4	8.3	7.6	7.5	7.8	7.7	7.6	7.2	7.2		
CGP 6140 1.25 × 10^-5^M (positive control)	2.5	0.3	0.0	0.0	0.0	0.0	0.0	0.0	0.0	100.0 (<0.001)	67.4 (0.0032)
Rifampicin 5 × 10^-5^M	8.0	6.6	2.1	0.4	0.4	0.0	0.0	0.0	0.0	100.0 (<0.001)	67.4 (<0.001)
Erythromycin 5 × 10^-5^M	8.4	8.5	8.0	7.6	7.7	7.6	7.4	7.5	7.4	0.0 (0.539)	0.0 (0.210)
Oxytetracycline 5 × 10^-5^M	8.0	8.1	7.7	7.5	7.6	7.5	7.1	6.4	6.9	4.2 (0.360)	0.0 (0.995)
Isoniazid 5 × 10^-5^M	8.2	8.2	7.7	8.0	7.7	7.3	6.8	5.7	5.2	27.8 (0.010)	35.5 (0.074)
Isoniazid 5 × 10^-5^M + Rifampicin 5 × 10^-5^M	7.8	8.2	5.0	4.0	2.7	0.8	0.0	0.0	0.0	100.0 (<0.001)	71.7 (0.002)
Trimethoprim 5 × 10^-5^M	8.4	8.4	7.9	7.6	7.6	8.0	7.5	7.0	7.0	2.8 (0.549)	0.0 (0.319)
Sulphamethoxazole 5 × 10^-5^M	8.1	8.3	8.4	7.6	7.6	8.0	7.5	7.3	7.1	1.4 (0.848)	0.0 (0.998)
Trimeth. 5 × 10^-5^M + Sulphamethox. 5 × 10^-5^M	8.5	8.4	8.0	7.5	6.8	7.5	7.1	7.4	6.9	4.2 (0.449)	0.0 (0.828)
Clofazimine 1.25 × 10^-5^M	7.7	6.5	5.2	3.5	4.3	3.5	3.2	0.8	0.0	100.0 (<0.001)	77.4 (<0.001)
Clofazimine 3.13 × 10^-6^M	8.2	7.0	4.5	5.5	5.0	4.8	3.4	1.2	0.8	88.9 (<0.001)	53.3 (0.028)

The effects of exposure to a range of antibiotics and antibiotic combinations on the viability of *Onchocerca gutturosa *adult males in long-term (40 day) *in vitro *culture. Viability was assessed by measuring worm motility scores throughout the trial and by MTT colorimetry on day 40.

Worm viability was measured by the motility levels and MTT colorimetry. Motility scores were assessed on an inverted microscope on a scale of 0 (immotile) to 10 (maximum) [22] at regular intervals up to 40 days. The biochemical evaluation of worm viability was carried out by MTT/formazan colorimetry on day 40. In this assay, the yellow compound MTT [3-(4,5-dimethylthiazol-2-yl)-2,5-diphenyltetrazolium bromide] is reduced by the mitochondrial enzyme succinate dehydrogenase of living tissues to produce the blue precipitate MTT formazan [24,25]. Single intact worms were placed in each well of a 48-well plate (Falcon, UK) containing 0.5 ml of 0.5 mg/ml MTT (Sigma, UK) in phosphate buffered saline, and incubated for 30 minutes at 37°C. The worms were then transferred to separate wells of a 96 well plate, each containing 200 μl of dimethyl sulphoxide to solubilize the formazan. After one hour the plate was gently agitated to disperse the colour evenly and the absorbance value (optical density) of the resulting formazan solution was determined at 490 nm on an ELISA reader. Inhibition of formazan formation is correlated with worm damage or death. The motility and MTT assay results were expressed as a mean percentage reduction compared with untreated control values at day 40. Comparisons of test groups to untreated controls for both motility levels and MTT colorimetry on day 40 were carried out using a 2-sample t-test.

### Contribution of *Wolbachia *to the MTT reduction assay

Since the *B. malayi *and *Drosophila melanogaster Wolbachia *genomes contain genes for succinate dehydrogenase [30], the mosquito cell-line C6/36 was used to investigate the contribution of *Wolbachia *to the MTT reduction assay. C6/36 is derived from *Aedes albopictus *larvae and is not naturally infected with *Wolbachia*. However, it can be easily infected with *Wolbachia *from the cell-line Aa23, which is derived from *A. albopictus *eggs and is naturally infected with *Wolbachia pipientis *[31]. The viability of neither cell is affected by either the presence or absence of the bacteria, since, unlike nematodes, insects do not have a mutualistic association with their *Wolbachia*. To infect C6/36 cells, medium from a confluent culture of Aa23 cells was collected, filtered through a 0.8 μm syringe filter to remove any whole mosquito cells, and placed into a flask of confluent C6/36 cells from which the medium had been removed. The flask was agitated gently at room temperature for 30 minutes before being incubated at 37°C in an atmosphere of 5% CO_2 _in air. Infection of the cells with the bacteria was confirmed by IFAT [17]. Hereafter the infected cells will be termed C6/36 Wp. C6/36 and C6/36 Wp were grown in L-15 Leibovitz medium (Gibco) supplemented with 2 mM L-glutamine, 1% non-essential amino acids, 2% tryptose phosphate broth and 5% foetal calf serum.

MTT reduction was compared with C6/36 and C6/36 Wp cells each with and without tetracycline treatment (20 μg/ml for up to four weeks, with medium changed every 3 days), with at least four repeats of 250 000 cells in each group. The media were removed and the cells washed and pelleted, then MTT was added to give a final concentration of 0.5 mg/ml in phosphate buffered saline. After an incubation of 1 hour at 37°C with 5% CO_2_, the cells were washed and pelleted, and 200 μl dimethyl sulphoxide was added to release the blue formazan reduction product. The optical density of the samples was measured at 490 nm and the results expressed as mean optical density (± S.D.). Student's *t*-test was used to compare differences between two groups, with *P *values of < 0.05 considered to be significant.

## Results

### *In vitro *drug screen

The motility scores and percentage inhibitions of MTT reduction by all the tested compounds are shown in Tables 1, 2 and 3. The positive control, CGP 6140, had a very rapid onset of action, with reductions in worm motility even on the first day after initiation of treatment. No worm movement was observed after day 15, and by day 40 there was a 67.4 to 83.3 inhibition in formazan formation compared to untreated controls. In contrast, the average motility score of the untreated control worms in each experiment ranged from 4.9 to 7.2 at day 40 (Tables 1, 2 and 3).

**Figure 1:**
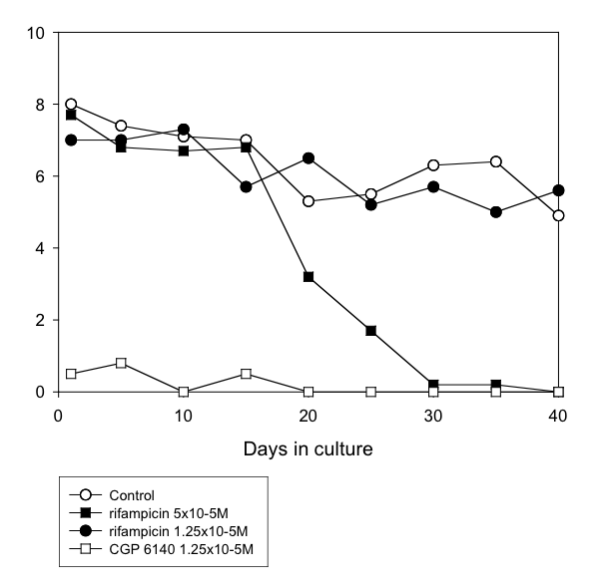
**Mean motility score of *O. gutturosa *adult males *in vitro *exposed to rifampicin. **Control, ○; rifampicin 5 × 10^-5^M, ■ rifampicin 1.25 × 10^-5^M, ●; CGP 6140 1.25 × 10^-5^M, □ (positive control). (Data from Table 1).

Several anti-mycobacterial drugs were tested. Of these, rifampicin (5 × 10^-5^M) was the most effective, completely inhibiting motility by day 40 (Table 1 and Fig. 1) or day 25 (Table 3). Clofazimine (1.25 × 10^-5^M and 3.13 × 10^-6^M) gradually affected worm motility so that, by day 40, there were 100% and 88.9% reductions in motility, and 77.4 and 53.3 inhibition of MTT reduction, with the higher and lower concentrations, respectively (Table 3 and Fig. 4). Ethambutol (5 × 10^-5^M) was less effective, giving 44.9% and 73% reductions in motility and MTT formazan formation, respectively (Table 1 and Fig. 5). However, the other agents with activity against *Mycobacterium *species showed limited (pyrazinamide, isoniazid) or no (dapsone) activity against the worms (Tables 1 and 3). Also, the addition of isoniazid (5 × 10^-5^M) to rifampicin (5 × 10^-5^M) did not improve the efficacy of the latter (Table 3).

**Figure 2:**
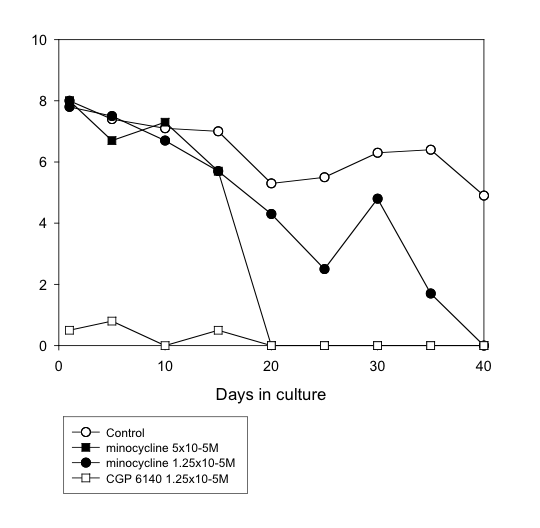
**Mean motility score of *O. gutturosa *adult males *in vitro *exposed to minocycline. **Control, ○; minocycline 5 × 10^-5^M, ■; minocycline 1.25 × 10^-5^M, ●; CGP 6140 1.25 × 10^-5^M, □ (positive control). (Data from Table 1).

**Figure 3:**
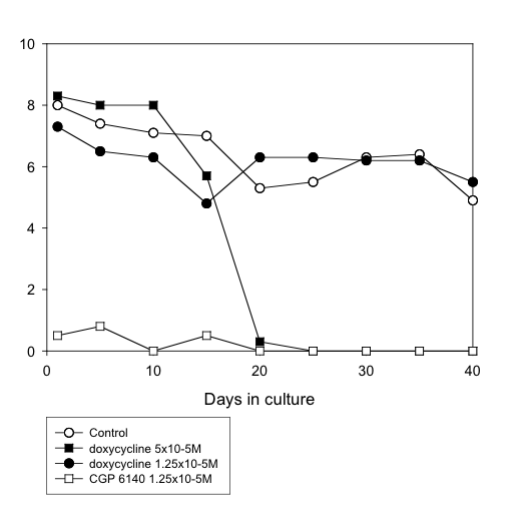
**Mean motility score of *O. gutturosa *adult males *in vitro *exposed to doxycycline. **Control, ○; doxycycline 5 × 10^-5^M, ■; doxycycline 1.25 × 10^-5^M, ●; CGP 6140 1.25 × 10^-5^M, □ (positive control). (Data from Table 1).

**Figure 4:**
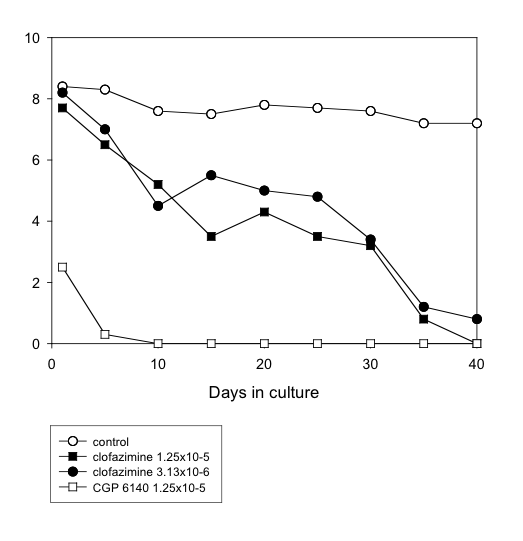
**Mean motility score of *O. gutturosa *adult males *in vitro *exposed to clofazimine. **Control, ○; clofazimine 1.25 × 10^-5^M, ■; clofazimine 3.13 × 10^-6^M, ●; CGP 6140 1.25 × 10^-5^M, □ (positive control). (Data from Table 3).

**Figure 5:**
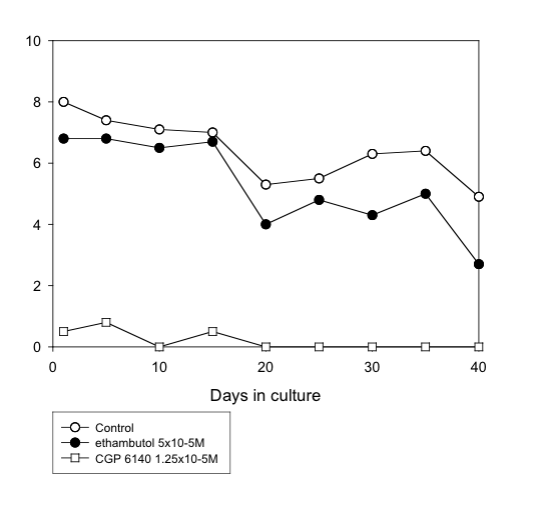
**Mean motility score of *O. gutturosa *adult males *in vitro *exposed to ethambutol. **Control,○; ethambutol 5 × 10^-5^M, ●; CGP 6140 1.25 × 10^-5^M, □ (positive control). (Data from Table 1).

Doxycycline was effective (100% reduction in motility by day 25, 93% inhibition of formazan formation at day 40) at a concentration of 5 × 10^-5^M, but showed no activity at 1.25 × 10^-5^M (Fig. 3). However, minocycline was filaricidal at both of these concentrations (Fig. 2). Intermediate activity was shown by norfloxacin, ciprofloxacin, vancomycin and gentamycin (all at a concentration of 5 × 10^-5^M). The drugs that did not have a filaricidal effect at 5 × 10^-5^M included erythromycin, oxytetracycline, trimethoprim and sulphamethoxazole (these two either alone or in combination).

Two compounds, triclosan and cerulenin, were toxic to the monkey kidney cell feeder layer, so it was not possible to conclude if they had an anti-*Onchocerca *effect, since a viable cell layer is essential to the long-term survival of the worms.

### Contribution of *Wolbachia *to the MTT reduction assay

**Figure 6:**
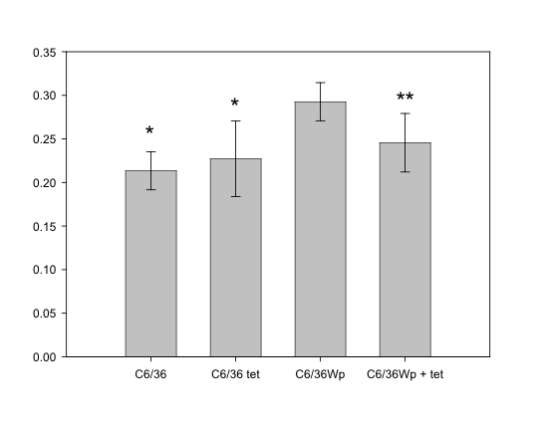
***Wolbachia *contribute to metabolic activity as measured by the MTT reduction assay **C6/36 and C6/36 Wp were incubated in medium or medium with tetracycline 20 μg/ml for four weeks before the MTT reduction assay was performed. Bars represent means of five repeats each of 250 000 cells (± S.D.). Values that are significantly lower than those of C6/36 Wp are denoted by * (*P *= 0.000) and ** (*P *= 0.03).

C6/36 cells with and without *W. pipientis *infection were incubated in medium or medium plus tetracycline for two or four weeks before being analysed by the MTT reduction assay. This assay showed that infection with *Wolbachia *did contribute to extra metabolic activity in the cells, since C6/36 Wp had significantly higher absorbance readings than C6/36 (*P *= 0.017 after two weeks' incubation; *P *= 0.000 after four weeks' incubation, Fig. 6). However, in C6/36 Wp treated with tetracycline for two to four weeks the absorbance was no different from C6/36 with tetracycline (*P *= 0.269 at two weeks; *P *= 0.475 at four weeks). At neither time-point did uninfected C6/36 treated with tetracycline show a significant difference from those not treated (*P *= 0.958 at two weeks; *P *= 0.543 at four weeks, Fig. 6), indicating that the tetracycline itself had no effect in reducing the metabolic activity.

## Discussion

The results presented here confirm that *O.gutturosa *males provide a suitable *in vitro *screen for slow-acting antibiotic drugs with macrofilaricidal activity. Male *O.gutturosa *are smaller than *O.volvulus *males but contain an almost identical *Wolbachia*/nematode ratio (H.F. McGarry and M.J. Taylor, unpublished observation) indicating that they are a suitable model for human onchocerciasis.

The gradual reduction in motility (as with clofazimine) or a delay before effects were seen (rifampcin, doxycycline, minocycline) is consistent with activity against the endosymbionts, in contrast to the direct and rapid effects on worm viability of the positive control, Amocarzine. Interestingly, with rifampcin, doxycycline, minocycline and ethambutol, the most rapid decline in worm motility occurred between 15 and 20 days of treatment. This period of treatment may be sufficient to inhibit *Wolbachia*-dependent processes, such as inhibition of protein synthesis (which is the mode of action of tetracyclines), with a resultant deterioration in nematode health before a decline in bacterial numbers is observable. As such, this *O. gutturosa *system is likely to be more sensitive than one that utilises *Wolbachia*-infected mosquito cell lines, which relies on direct observation for the presence or absence of bacteria [27,32].

Currently, doxycycline (200 mg) administered daily for three weeks is the shortest effective regimen that has been tried against a human filarial infection; when followed by standard anti-filarial chemotherapy, this treatment resulted in prolonged reductions in microfilaraemia and in *Wolbachia *numbers in the microfilariae, but was not macrofilaricidal [20] and is similar to the timeframe after which effects were observable *in vitro*.

Rifampicin has previously been shown to be very effective against *Wolbachia *[6,27,32,33], which was confirmed in this *in vitro *antifilarial assay. Rifampicin interferes with nucleic acid synthesis by combining with and inhibiting the RNA-polymerase of bacteria and is bactericidal. The anti-leprosy agent clofazimine also demonstrated good activity against *O. gutturosa*, and is also bactericidal. Anti-tuberculosis and -leprosy therapies may have benefits at the population level by reducing the prevalence of filariasis [34]. However, the other drugs tested that are used against *Mycobacterium *species (ethambutol, dapsone, pyrazinamide, isoniazid) showed little anti-*Onchocerca *activity.

The tetracycline doxycycline was filaricidal in this drug screen, confirming previous findings that it has anti-*Wolbachia *effects [27, 33, 35]. This drug has also been used in human filarial infections, in which it has resulted in a prolonged loss of microfilariae and lack of embryogenesis [10,11,14], and has recently been shown to be macrofilaricidal against *W. bancrofti *[13]. In the present work minocycline was even more effective than doxycycline and warrants further investigation *in vivo*. This result agrees with our unpublished work (S. Townson) that minocycline is more active than doxycycline against *O. lienalis *microfilariae in mice. Minocycline has the advantage over doxycycline of inducing less phototoxicity. However, oxytetracycline showed no anti-*Wolbachia *activity, in contrast to previous reports [6,9,32]; it is not clear why there were contradictory findings. The lack of activity by other compounds (erythromycin, trimethoprim and sulphamethoxazole) was consistent with previous findings [27, 36].

Filarial nematodes cultured *in vitro *often succumb to drug treatments more readily than they do *in vivo*. The system described here provides a relatively easy and inexpensive way to perform a primary or secondary screen of compounds for anti-filarial/*Wolbachia *activity, which would then be followed by *in vivo *experiments. Antibiotics are slow-acting against filarial nematodes, as seen in the present study and in field trials (for example, [12,13]); an advantage of the male *O. gutturosa *culture system is that it can be maintained for at least 40 days. In the present study the effects of the compounds has not been directly related to their activity against *Wolbachia *and it is possible that they also had direct anti-nematodal activity. Studies on the dynamics of the loss of the bacteria from worms are currently underway using quantitative polymerase chain reaction and/or immunohistology in the screening system.

The presence of *Wolbachia *made a significant contribution to the MTT assay in C6/36 cells as would be predicted from the presence of succinate dehydrogenase as determined by genomic annotation. Thus partial reduction in formazan formation in MTT assays could reflect either the loss of bacteria and retention of worm viability or *vice versa*. Should an *in vitro *screen for specific activity against either the bacteria or worm be required then alternative markers of viability may need to be developed [37].

## Conclusion

This study has demonstrated that the in *vitro screen *for macrofilaricidal activity, which uses the culture of male *O. gutturosa*, can be successfully extended and is also valid for the screening of antibiotic compounds with potential anti-*Wolbachia*/filarial activity. This system can be used for long-term screening, in this case for 40 days. Rifampicin and doxycycline were two of the most active antibiotics tested in this screen, in agreement with previous findings. However, a new finding was that minocycline was more quickly and completely effective than either of these compounds. It was also found that *Wolbachia *contribute to the MTT formazan formation which is used as a marker of filarial worm viability, suggesting that bacteria contribute directly to the metabolic activity of the nematode and that it may be necessary to reassess alternative indicators of worm and or bacterial viability.

## Abbreviations

MTT, 3-(4,5-dimethylthiazol-2-yl)-2,5-diphenyltetrazolium bromide; MDA, mass drug administration.

## Competing interests

The author(s) declare that they have no competing interests.

## Authors' contributions

STo designed the antibiotic screen experiments, analysed the results and advised on the manuscript preparation. STo and STa performed the antibiotic screen experiments. HFM analysed results, performed statistical analysis and prepared the manuscript. GLE performed the C6/36 MTT analyses. MJT designed the C6/36 MTT assay experiments, analysed the results and advised on the manuscript preparation.
